# Flows of Linear Polymer Solutions and Other Suspensions of Rod-like Particles: Anisotropic Micropolar-Fluid Theory Approach

**DOI:** 10.3390/polym13213679

**Published:** 2021-10-25

**Authors:** Vladimir Shelukhin

**Affiliations:** 1 Lavrentyev Institute of Hydrodynamics, 630090 Novosibirsk, Russia; shelukhin@list.ru; 2 Mathematical Department, Novosibirsk State University, 630090 Novosibirsk, Russia

**Keywords:** suspension, rodlike particles, rheology, micropolar fluids, anisotropy, hysteresis

## Abstract

We formulate equations governing flows of suspensions of rod-like particles. Such suspensions include linear polymer solutions, FD-virus, and worm-like micelles. To take into account the particles that form and their rotation, we treat the suspension as a Cosserat continuum and apply the theory of micropolar fluids. Anisotropy of suspensions is determined through the inclusion of the microinertia tensor in the rheological constitutive equations. We check that the model is consistent with the basic principles of thermodynamics. In addition to anisotropy, the theory also captures gradient banding instability, coexistence of isotropic and nematic phases, sustained temporal oscillations of macroscopic viscosity, shear thinning and hysteresis. For the flow between two planes, we also establish that the total flow rate depends not only on the pressure gradient, but on the history of its variation as well.

## 1. Introduction

There is a class of complex fluids which can be considered as suspensions of rod-like particles. Examples include linear polymer solutions, worm-like micelles, FD-virus, liquid crystals, etc. Such a class enjoys interesting properties like anisotropy [1], gradient and vorticity banding [2,3,4], shear banding instabilities [5], transition between isotropic and nematic phases [6], and cluster formation [7,8].

Here, we formulate a new mathematical model which is good for concentrated suspensions and show that it predicts anisotropy and some other properties of suspensions of rod-like particles. To this end, we study Poiseuille-like shear flows. The practice of pumping oil in pipelines shows that the total oil flux can depend not only on the pressure gradient, but on the history of pumping as well [9]. We establish that the developed model captures such an effect and show its relationship with the hysteresis phenomenon.

Studies of rodlike particles flow in fluids go back to Jeffrey’s work on interactions of a floating isolated ellipsoid with unbounded linear shear fluid flow [10]. It turns out that such a particle periodically rotates in Jeffrey’s orbits, which depend on the geometry of the particle and its initial orientation. Jeffery’s approach was developed further in a number of kinematic models [11,12], which include equations both for particle mass centre and for the direction vector with the help of a third rank shape tensor. Available experiments [13,14] confirmed applicability of the generalized Jeffery equations. Such an approach formed basis for extensions accounting for rod–rod interactions [15,16] and for the prediction fibre alignment distributions in moulded parts [17]. Equations proposed in [18] also allow for governing particles motion in a simplified situation where the rod orientation is restricted to the plane spanned by the direction of shear and the direction of gravity.

In a number of studies, the search for the rheology of suspensions of rodlike particles is reduced to establishing the relationship between stress and rate of strain in shear flows. In [19], starting from experiments with FD-viruses, it was studied how viscosity depends on concentration, shear rate and ionic strength. An expression for viscosity was derived in [20] with the use of friction coefficients parallel and perpendicular to the rod axis. We refer the reader to detailed description of viscosity representation formulas to [1,19,21]; the viscosity dependence on shear rate is also discussed there.

Our approach is different. We use methods of mechanics of continua by applying conservation laws only and not involving the concept of particle direction. To take into account particle rotation and form, we apply the theory of micropolar fluids, which allows for particle microinertia [22]. According to this theory, which is a part of rational mechanics, any infinitesimal volume contains sufficiently many particles. This is why such an approach is applicable for suspensions with a high concentration of particles. As is proved within the micropolar fluid theory in [23], it is due to particle rotation that the Segre–Silberberg effect occurs. Such an effect is known as a tubular pinch phenomenon, stating that particles tend to migrate towards a concentric annular region for the laminar flow of neutrally buoyant dilute suspension of rigid spheres through a circular tube [24]. There is one more effect caused by particle rotation and rotational diffusion. This is the separation of particles when flowing between two concentric rotating cylinders [25].

The micropolar fluid theory allows for intrinsic rotations and micro-inertia thanks to the concept of the Cosserat continuum where each material point is treated as a rigid body [26]. We formulate anisotropic constitutive law by including the micro-inertia tensor into stress/rate of strain relationships. Such an idea of anisotropy was first formulated in [27]. In a great number of papers, rotation of the particles is neglected and the anisotropy is taken into account by using the differences of normal stresses [28].

In the micro-polar fluid theory allowing for internal spins, stress tensor loses symmetry, couple stress appears, and the angular momentum equation should be included into conservation laws. Formulation of rheological constitutive laws in the present paper involves introduction of new viscosities both relative to the Cauchy stress tensor and to the couple stress tensor. Skew-symmetric and anisotropic viscosities are introduced in addition to the common shear viscosity, which we call here symmetric viscosity. While there are experiments [29] and theories [25] to determine the skew-symmetric viscosity, the question of measuring the anisotropic viscosity remains open. We cannot quantitatively confirm our equations by three-dimensional experiments, since the calculations were carried out on the basis of one-dimensional flows. However, we prove that it is precisely due to the anisotropic viscosity that these equations capture such effects as hysteresis, shear gradient banding instability and phase transition.

The flow of short polymer chains in the channels can be regarded as an example of the applicability of the method outlined in this work. The flow of such polymers between graphite surfaces is studied in [30] by the molecular dynamic simulation technique. It is established there that the polymer chains exhibit preferential alignment of oligomers parallel to the surfaces with increasing shear rate. Though in the present paper it is assumed that rods lie in the plane orthogonal to the bounding planes, we predict like in [30] that the apparent viscosity shows an oscillatory behaviour and its variation versus the shear rate corresponds to the shear thinning phenomenon. We perform calculations of the simple flow depending on one variable only; nevertheless, we capture appearance and instability of the nematic phase. More complicated phase transition was addressed in [31] for colloidal suspensions in water; the nucleation of a kagom lattice from solution was detected.

The goal of Section 2 is to remind foundations of the micropolar fluid theory and formulate conservation and constitutive laws obeying the basic principles of thermodynamics. In Section 3, we derive equations for one-dimensional Poiseuille-like shear flows. Finally, in Section 4, we perform calculations explaining different phenomena. In addition to anisotropy, the calculations predict gradient banding instability, phase transition between isotropic and nematic phases, sustained temporal oscillations of macroscopic viscosity, shear thinning and hysteresis. For the flow between two planes, we also establish that the total flow rate depends not only on the pressure gradient, but on the history of its variation as well.

## 2. Anisotropic Micropolar Fluids

We remind basic notions of the micro-polar fluid theory. Given a material point labelled by the Lagrangian coordinate-vector ξ, the position vector x(t,ξ) at the time instant *t* in the three dimensional Euclidean space jointly with orthogonal director-vectors $d_{i}\left(t,\xi \right)$, i=1,2,3, are assigned to such a point to treat it as a rigid body. Orientation of $d_{i}$ is controlled by an orthogonal tensor Q(t,ξ):

$$d_{i}\left(t,\xi \right)=Q\left(t,\xi \right)d_{i}\left(0,\xi \right),\;Q^{*}Q=QQ^{*}=I.$$

Here, *I* is the identity matrix with the elements $\delta_{j}^{i}$, $Q^{*}$ is the adjoint matrix, ${\left(Q^{*}\right)}_{ij}=$ $Q_{ji}$. The skew-symmetric tensor $\Omega \left(t,x\right)=Q_{t}Q^{*}$ defines the particle’s rotation with the angular velocity

$$\omega \left(t,x\right)=e_{i}\times \left(\Omega e_{i}\right)/2=\epsilon :\Omega /2,\;{\left(\Omega a\right)}_{i}=\Omega_{ij}a_{j}\;\forall \;a\in R^{3},$$

where ${\left\{e_{i}\right\}}_{1}^{3}$ is an orthonormal basis in $R^{3}$ and ϵ is the Levi–Civita third order tensor,

$$\varepsilon \left(a,b,c\right)=a\cdot \left(b\times c\right),\;e_{i}\times e_{j}=\epsilon_{sij}e_{s},\;\epsilon_{sij}=\varepsilon \left(e_{s},e_{i},e_{j}\right),\;{\left(\epsilon :\Omega \right)}_{i}=\epsilon_{ijk}\Omega_{jk}.$$

Given the velocity field v(t,x)= $x_{t}\left(t,\xi \right)$, we introduce the rate of strain tensors [22].

B=∇v−Ω,A=∇ω,

where ${\left(\nabla v\right)}_{ij}=\partial v_{i}/\partial x_{j}$. Observe that both *B* and *A* are objective relative to the change of frame of references.

Let ρ, s, *T* and *N* stand for the mass density, the specific internal spin, the Cauchy stress tensor and the angular moment tensor, respectively. We introduce the material derivative $\dot{\rho }$ (or dρ/dt) related to the velocity field v as follows

(1) $$\dot{\rho }=\frac{\partial \rho }{\partial t}+v_{i}\frac{\partial \rho }{\partial x_{i}}\;\mathrm{or}\;\dot{\rho }=\frac{\partial \rho }{\partial t}+\left(v\cdot \nabla \right)\rho .$$

Conservation laws of mass, momentum and angular momentum are given by the equations

(2) $$\dot{\rho }+\rho div\;v=0,$$

(3) $$\rho \;\dot{v}=div\;T+\rho \;f,$$

(4) $$\rho \;\dot{s}=div\;N-\epsilon :T+\rho \;l,$$

where f is the mass force density, l is the mechanical couple density, and

$${\left(div\;T\right)}_{i}=\partial T_{ij}/\partial x_{j}.$$

Observe that the stress tensor *T* is not symmetric. Given an orthonormal basis ${\left\{e_{i}\right\}}_{1}^{3}$, the vector

$$t=e_{i}\times \left(T\cdot e_{i}\right)=\varepsilon :T$$

does not depend on the choice of the basis and it is a stress symmetry defect measure in the sense that the equality t=0 implies $T^{*}=T$ and vice versa. By the definition of t, we have the formula

(5) t·ω=T:Ω.

The internal specific spin is defined by the formula s= Jω, where the symmetric inertia tensor $J[{\mathrm{cm}}^{2}]$ obeys the identity [22]

(6) $$\dot{J}-\Omega J+J\Omega =0.$$

Before proceeding to constitutive laws, we address the thermodynamics issue. Given a specific internal energy *e*, the total energy E=e+v·v/2+s·ω/2 satisfies the equation [32]

(7) $$\rho \;\dot{E}=div\;\left(T^{*}v+N^{*}\omega -q\right)+\rho \;f\cdot v+\rho \;l\cdot \omega ,$$

where q is the heat flux obeying the Fourier law q=− ϰ∇θ, with ϰ standing for the heat conductivity. Generally, internal energy *e* depends on ρ, η and *J*, e=e(ρ,η,J), where η is the specific entropy. It is common knowledge that absolute temperature and pressure are defined by the derivatives θ=∂e/∂η, $p=\rho^{2}\partial e/\partial \rho$ respectively [33]. We calculate that

$$\dot{e}=e_{\rho }\dot{\rho }+e_{\eta }\dot{\eta }+\nabla_{J}e:\dot{J},\;\mathrm{where}\;\nabla_{J}e:\dot{J}=\left({\dot{J}}_{ij}\frac{\partial }{\partial J_{ij}}\right)e.$$

From the rheological point of view, the internal energy e(ρ,η,J) should be an isotropic function of *J*. Hence, $\nabla_{J}e$ is also an isotropic function of *J*; it implies that [34]

(8) $$\nabla_{J}e=\alpha_{0}I+\alpha_{1}J+\alpha_{2}J^{2},$$

where the scalar functions $\alpha_{i}$ depend on invariants of *J*. Now, it follows from (6) and (8) that $\nabla_{J}e:\dot{J}=0.$

We use Equation (6) to calculate that

$$\frac{d}{dt}\left(s\cdot \omega \right)=\dot{s}\cdot \omega +s\cdot \dot{\omega }=2\dot{s}\cdot \omega .$$

Hence,

(9) $$\rho \dot{E}=\frac{p}{\rho }\dot{\rho }+\rho \theta \dot{\eta }+\rho \dot{v}\cdot v+\rho \dot{s}\cdot \omega =-p\mathrm{div}\;v+\rho \dot{v}\cdot v+\rho \dot{s}\cdot \omega .$$

Multiplying Equations (3) and (4) by v and ω, respectively, we arrive at the energetic equality

(10) $$\rho \;v\cdot \dot{v}+\rho \;\omega \cdot \dot{s}=\mathrm{div}\left(T^{*}v+N^{*}\omega \right)-T:B-N:A+\rho \;f\cdot v+\rho \;l\cdot \omega$$

With *S* standing for the viscous part of the stress tensor *T*, we write the representation formula T=−pI+S. Hence, T:B=−pdivv+ S:B, where $T:B=T_{ij}B_{ij}$. Now, it follows from (7), (9) and (10) that the entropy equation

(11) $$\rho \dot{\eta }+\mathrm{div}\left(\frac{q}{\theta }\right)=\frac{R}{\theta },$$

holds, with the function

$$R=S:B+N:A+\frac{{\varkappa |\nabla \theta |}^{2}}{\theta }$$

standing for the entropy production. The second law of the thermodynamics R≥0 can be formulated as

(12) S:B+N:A≥0.

In what follows, we use the notations

$$B_{s}=\frac{B+B^{*}}{2},\;B_{a}=\frac{B-B^{*}}{2}$$

for the symmetric and skew-symmetric parts of *B*. We formulate anisotropic constitutive laws as follows:

(13) $$S=2\mu_{s}B_{s}+2\mu_{a}B_{a}+2\mu_{an}\sigma^{2}JB,\;N=\frac{2\nu }{\sigma^{2}}A+2\nu_{an}AJ,$$

where $\mu_{s},\mu_{a},\mu_{an},\nu ,\nu_{an}\left[g/(\mathrm{cm}\cdot s)\right]$ are the viscosities and $\sigma [{\mathrm{cm}}^{-1}]$ is the specific particles surface area. The first rheological equation in (13) suggests that the contributions of the symmetric part $B_{s}$ and skew-symmetric part $B_{a}$ of the rate of strain tensor *B* into local stress state are different. The fact that both *S* and *N* depend on *J* implies anisotropy. Such an approach was first formulated in [22]. Observe that the objectivity of the *S* and *N* results form the objectivity of *B*, *A* and *J* [22].

Due to the symmetry of *J*, one can verify that

$$JB:B=\sum_{1}^{3}\lambda_{j}{\left|B^{*}e_{j}\right|}^{2},$$

where $e_{j}$ and $\lambda_{j}$ are the eigenvectors and the eigenvalues of *J*. Observe that $\lambda_{j}\geq 0$ provided each suspension particle enjoys an axis of rotational symmetry. For such suspensions, we find that

$$S:B=2\mu_{s}B_{s}:B_{s}+2\mu_{a}B_{a}:B_{a}+2\mu_{an}\sigma^{2}\sum_{1}^{3}\lambda_{j}{\left|B^{*}e_{j}\right|}^{2}\geq 0.$$

Similarly, one can verify that

$$AJ:J=\sum \lambda_{j}{\left|Ae_{j}\right|}^{2}.$$

Thus, the constitutive laws (13) satisfy the thermodynamic restriction (12), provided the suspension particles are axially symmetric.

## 3. Poiseuille Flows

**Figure 1:**
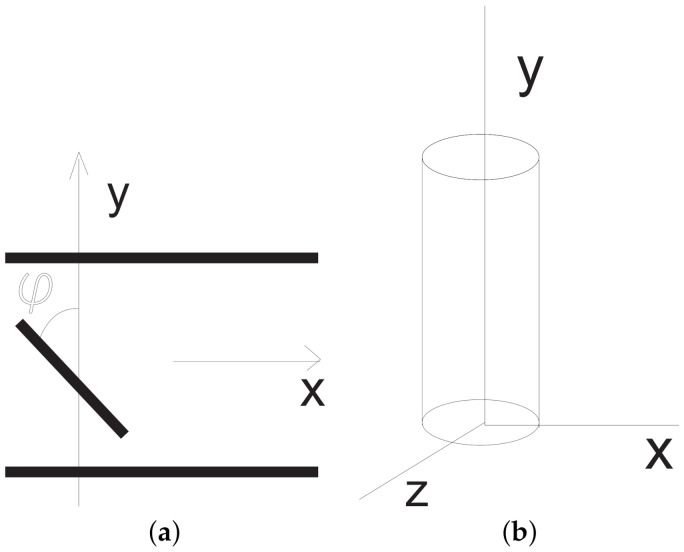
(**a**) Schema of particle’s position in one-dimensional flows. (**b**) The cylinder approximation of the rod-like particle.

We consider one-dimensional flows along the horizontal *x*-axis in the vertical layer |y|<H between two parallel planes under the prescribed pressure gradient ∇p= $\left(p_{x},0,0\right)$, $p_{x}\left(t\right)<0$, Figure 1a. In this case, $v_{2}=v_{3}=0$, $v_{1}=v\left(y,t\right)$. We assume that the suspension particles are the rods of the same size; they lie in the plane z=0 and rotate around the *z*-axis. Hence, ω=(0,0,ω).

Let us describe the micro-inertia tensor *J*. First, we consider a cylinder $V_{0}$ stretched along the *y*-axis with the height *h* and the radius *r*, Figure 1b. By definition, the inertia tensor $J(V_{0})$ of $V_{0}$ is given by the formula

$$J\left(V_{0}\right)=\int_{V_{0}}{\left|\zeta \right|}^{2}\cdot I-\xi ⊗\xi \;d\xi \;\mathrm{or}\;J_{ij}\left(V_{0}\right)=\int_{V_{0}}{\left|\xi_{k}\right|}^{2}\delta_{ij}-\xi_{i}\xi_{j}\;d\xi ,$$

where *I* is the identity matrix and a⊗b stands for the tensor product of two vectors a and b, ${\left(a⊗b\right)}_{ij}=$ $a_{i}b_{j}$. Calculations reveal that

$$J\left(V_{0}\right)=\left(\begin{array}{ccc} r^{2}/4+h^{2}/3 & 0 & 0\\ 0 & r^{2}/2 & 0\\ 0 & 0 & r^{2}/4+h^{2}/3 \end{array}\right).$$

In the limit as r→0, we obtain the inertia tensor of the rod particle stretched along the *y*-axis:

$$J^{0}=\lim_{r\to 0}J\left(V_{0}\right)=j_{0}\left(\begin{array}{ccc} 1 & 0 & 0\\ 0 & 0 & 0\\ 0 & 0 & 1 \end{array}\right).\;j_{0}=h^{2}/3.$$

Let J(V) be the inertia tensor of the cylinder *V*, which is produced by rotation of $V_{0}$ around the *z*-axis by the angle φ counted from the axis *y* counter-clockwise, see Figure 1a. By definition of the spin s, we find that

$$s=J\left(V\right)\cdot \omega =\int_{V}x\times \left(\omega \times x\right)\;dx=Q_{\varphi }J^{0}Q_{\varphi }^{*}\cdot \omega ,$$

where $Q_{\varphi }$ is the orthogonal matrix such that

(14) $$\Omega ={\dot{Q}}_{\varphi }Q_{\varphi }^{*},\;\Omega \cdot h=\omega \times h\;\forall \;h,\;{\left(Q^{*}\right)}_{ij}=Q_{ji},\;$$

(15) $$Q_{\varphi }=\left(\begin{array}{ccc} \cos\varphi & -\sin\varphi & 0\\ \sin\varphi & \cos\varphi & 0\\ 0 & 0 & 1 \end{array}\right),\;\Omega =\left(\begin{array}{ccc} 0 & -\omega & 0\\ \omega & 0 & 0\\ 0 & 0 & 0 \end{array}\right),$$

with “dot” standing for the material derivative (1) related to the velocity vector v. Thus, $J\left(V\right)=Q_{\varphi }J\left(V_{0}\right)Q_{\varphi }^{*}$. In the limit as r→0, we obtain the inertia tensor J(φ) of the rod particle with the position angle φ:

(16) $$J\left(\varphi \right)=Q_{\varphi }J^{0}Q_{\varphi }^{*}=j_{0}\left(\begin{array}{ccc} \cos^{2}\varphi & \sin\varphi \cos\varphi & 0\\ \sin\varphi \cos\varphi & \sin^{2}\varphi & 0\\ 0 & 0 & 1 \end{array}\right),\;\frac{\partial \varphi }{\partial t}=\varphi_{t}=\omega .$$

Given an initial distribution of particle’s angles $\varphi_{0}\left(y\right)$, we denote the initial micro-inertia tensor by $J_{0}\left(y\right)=$ $J(\varphi_{0}\left(y\right))$:

(17) $${J|}_{t=0}=J_{0}.$$

For the described one-dimensional flows, the material derivative $\dot{J}$ reduces to the time derivative $J_{t}$. With the use of Equation (15), (6) can be written as follows:

(18) $$\frac{\partial }{\partial t}J_{11}=-2\omega J_{12},\;\frac{\partial }{\partial t}J_{12}=\omega \left(J_{11}-J_{22}\right),\;\frac{\partial }{\partial t}J_{22}=2\omega J_{12},$$

and $J_{ij}=0$ otherwise. Observe that

$$\frac{\partial J_{ij}}{\partial t}=\frac{\partial J_{ij}}{\partial \varphi }\frac{\partial \varphi }{\partial t}=J_{ij}^{\prime }\omega ,\;J_{ij}^{\prime }=\frac{\partial J_{ij}}{\partial \varphi }.$$

Hence, the system (18) is equivalent to

(19) $$J_{11}^{\prime }=-2J_{12},\;J_{12}^{\prime }=J_{11}-J_{22},\;J_{22}^{\prime }=2J_{12},\;J_{ij}{|}_{\varphi =\varphi_{0}}=J_{0}.$$

One can verify that the matrix *J* in (16) solves the system (19).

We calculate the rate of strain tensors and find that

(20) $$B=\left(\begin{array}{ccc} 0 & v_{y}+\omega & 0\\ -\omega & 0 & 0\\ 0 & 0 & 0 \end{array}\right),\;B^{*}=\left(\begin{array}{ccc} 0 & -\omega & 0\\ v_{y}+\omega & 0 & 0\\ 0 & 0 & 0 \end{array}\right),$$

$$B_{s}=\left(\begin{array}{ccc} 0 & v_{y}/2 & 0\\ v_{y}/2 & 0 & 0\\ 0 & 0 & 0 \end{array}\right),\;B_{a}=\left(\begin{array}{ccc} 0 & v_{y}/2+\omega & 0\\ -v_{y}/2-\omega & 0 & 0\\ 0 & 0 & 0 \end{array}\right),$$

$$j_{0}^{-1}JB=\left(\begin{array}{ccc} -\omega \cos\varphi \sin\varphi & \cos^{2}\varphi \left(v_{y}+\omega \right) & 0\\ -\omega \sin^{2}\varphi & \cos\varphi \sin\varphi (v_{y}+\omega ) & 0\\ 0 & 0 & 0 \end{array}\right),$$

(21) $$A=\left(\begin{array}{ccc} 0 & 0 & 0\\ 0 & 0 & 0\\ 0 & \omega_{y} & 0 \end{array}\right),\;j_{0}^{-1}AJ=\left(\begin{array}{ccc} 0 & 0 & 0\\ 0 & 0 & 0\\ \omega_{y}\cos\varphi \sin\varphi & \omega_{y}\sin^{2}\varphi & 0 \end{array}\right),$$

Let us denote

$$\varepsilon_{1}=\frac{\mu_{a}}{\mu_{s}},\;\varepsilon_{20}=\frac{\mu_{an}j_{0}\sigma^{2}}{\mu_{s}},\;\varepsilon_{30}=\frac{\nu_{an}j_{0}\sigma^{2}}{\nu },\;B^{0}=B_{s}+\varepsilon_{1}B_{a}+\varepsilon_{20}JB.$$

Calculations reveal that matrix $B^{0}$ is equal to

$$\left(\begin{array}{ccc} -\varepsilon_{20}\omega \cos\varphi \sin\varphi & \frac{v_{y}\left(1+\varepsilon_{1}+2\varepsilon_{20}\cos^{2}\varphi \right)}{2}+\omega \left(\varepsilon_{1}+\varepsilon_{20}\cos^{2}\varphi \right) & 0\\ \frac{v_{y}\left(1-\varepsilon_{1}\right)}{2}-\omega \left(\varepsilon_{1}+\varepsilon_{20}\sin^{2}\varphi \right) & \varepsilon_{20}\cos\varphi \sin\varphi \left(v_{y}+\omega \right) & 0\\ 0 & 0 & 0 \end{array}\right).$$

We consider incompressible fluids with the assumption ρ= const. For one-dimensional flows, the incompressibility condition divv=0 is satisfied automatically. Other conservation laws (3) and (4) become

(22) $$\varphi_{t}=\omega ,\;\rho v_{t}=-p_{x}+\frac{\partial S_{12}}{\partial y},\;\rho j_{0}\omega_{t}=\frac{\partial N_{32}}{\partial y}+S_{21}-S_{12}.$$

For one-dimensional flows, the constitutive laws (13) reduce to

(23) $$S_{ij}=2\mu_{s}B_{ij}^{0},\;N_{32}=2\frac{\nu }{\sigma^{2}}A_{32}+2\nu_{an}{\left(AJ\right)}_{32}.$$

Observe that

$$S_{21}-S_{12}=2\mu_{s}\left(B_{21}^{0}-B_{12}^{0}\right),\;B_{21}^{0}-B_{12}^{0}=-v_{y}\left(\varepsilon_{1}+\varepsilon_{20}\cos^{2}\varphi \right)-\omega \left(2\varepsilon_{1}+\varepsilon_{20}\right).$$

We formulate boundary conditions at |y|=H as follows:

(24) $$v=0,\;\omega =\frac{\alpha }{2}\nabla \times v,\;0\leq \alpha \leq 1.$$

The first condition in (24) states that velocity obeys the no-slip condition. The second condition in (24) has the meaning that the micro-rotation ω depends linearly on the macro-rotation ∇×v/2 [25].

Let *V* and *T* stand for the velocity and time reference values. We denote Ω=V/H and choose T=1/Ω. We introduce dimensionless variables as follows:

$$S^{\prime }=\frac{1}{2\mu_{s}\Omega }S,\;N^{\prime }=\frac{H\sigma^{2}}{2\nu \Omega }N,\;B^{\prime 0}=\frac{B^{0}}{\Omega },\;Re=\frac{H^{2}\rho \Omega }{\mu_{s}},$$

$$y^{\prime }=\frac{y}{H},\;v^{\prime }=\frac{v}{V},\;t^{\prime }=\frac{t}{T},\;\omega^{\prime }=\frac{\omega }{\Omega },\;\Pi =\frac{|p_{x}|H^{2}}{2V\mu_{s}},\gamma =\frac{\nu }{H^{2}\mu_{s}\sigma^{2}}.$$

In new variables, Equation (22) become

(25) $$\varphi_{t^{\prime }}=\omega^{\prime },\;\frac{Re}{2}v_{t^{\prime }}^{\prime }=\Pi +\frac{\partial S_{12}^{\prime }}{\partial y^{\prime }},\;\frac{Rej_{0}}{2H^{2}}\omega_{t^{\prime }}^{\prime }=\gamma \frac{\partial N_{32}^{\prime }}{\partial y}+S_{21}^{\prime }-S_{12}^{\prime }.$$

In what follows, we consider quasi-steady slow flows. Neglecting terms with small Reynolds numbers in (25), we arrive at the equations

(26) $$\varphi_{t^{\prime }}=\omega^{\prime },\;0=\Pi +\frac{\partial S_{12}^{\prime }}{\partial y^{\prime }},$$

(27) $$0=\gamma \frac{\partial }{\partial y^{\prime }}\left[\omega_{y^{\prime }}^{\prime }\left(1+\varepsilon_{30}\sin^{2}\varphi \right)\right]-\left[v_{y^{\prime }}^{\prime }\left(\varepsilon_{1}+\varepsilon_{20}\cos^{2}\varphi \right)+\omega^{\prime }\left(2\varepsilon_{1}+\varepsilon_{20}\right)\right],$$

where

$$S_{12}^{\prime }=\frac{v_{y^{\prime }}^{\prime }\left(1+\varepsilon_{1}+2\varepsilon_{20}\cos^{2}\varphi \right)}{2}+\omega^{\prime }\left(\varepsilon_{1}+\varepsilon_{20}\cos^{2}\varphi \right).$$

Because of the symmetry conditions

$$v^{\prime }\left(-y^{\prime },t^{\prime }\right)=v^{\prime }\left(y^{\prime },t^{\prime }\right),\;\omega^{\prime }\left(-y^{\prime },t^{\prime }\right)=-\omega^{\prime }\left(y^{\prime },t^{\prime }\right),\;\varphi \left(-y^{\prime },t^{\prime }\right)=\varphi \left(y^{\prime },t^{\prime }\right)$$

we consider flows only in the upper half-layer $0<y^{\prime }<1$. In such a case the initial and boundary conditions take the form

(28) $${\varphi |}_{t^{\prime }=0}=\varphi_{0}\left(y^{\prime }\right),\;v^{\prime }\left(1\right)=0,\;v_{y^{\prime }}^{\prime }\left(0\right)=0,\;\omega^{\prime }\left(1\right)=-0.5\alpha v_{y^{\prime }}^{\prime }\left(1\right),\;\omega^{\prime }\left(0\right)=0.$$

To perform numerical solution, one should fix the dimensionless parameters Π, $\varepsilon_{1}$, $\varepsilon_{20}$, $\varepsilon_{30}$, γ, α.

## 4. Results of Calculations

Here, we address the system (26)–(28) by applying the Wolfram Mathematica solver.

It is well known in many complex fluids that a shear banding effect occurs when applied shear stress is above some critical value [4,35,36]. Such a phenomenon is characterized by coexisting bands of different shear rates and /or viscosities. Depending on the directional alignment of the banded structure, there are two types of shear banding for suspensions of rod-like particles: gradient banding and vorticity banding [2,3,4]. In the case of gradient banding, the flow separates into bands of different shear rates along the gradient direction. With reference to the coordinate system of Figure 1a, *x* is the flow direction along the velocity vector v= (v,0,0), *y* is the gradient direction along which the flow has non-zero derivative ∂v/∂y. The *z*-axis is the vorticity direction along the non-zero macro-vorticity vector ∇×v.

**Figure 2:**
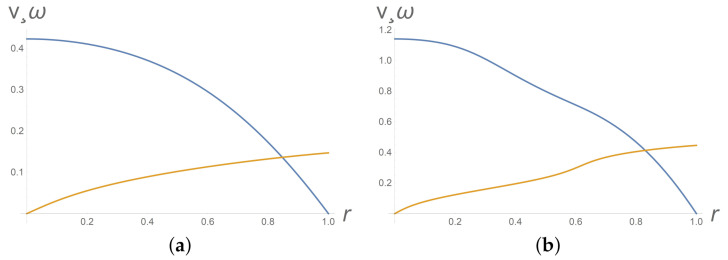
From top to bottom, profiles of the dimensionless velocity v(y) and dimensionless micro-spin ω(y) on the upper half-layer 0<y<1 at dimensionless time t=10 for dimensionless pressure gradient (**a**) Π=0.85 and (**b**) Π=2.85. Gradient banding development is observed at high pressure gradients (**b**).

The system (26)–(28) cannot be applied for description of the vorticity banding since the corresponding one-dimensional flow does not depend on the *z*-variable. However, calculations reveal that the system (26)–(28) can really capture the gradient banding. Figure 2 depicts appearance of gradient banding when shear stress increases; calculations are performed at t=10 for

(29) $$\varepsilon_{1}=1,\;\varepsilon_{20}=2,\;\varepsilon_{30}=2,\;\gamma =1.3,\;\alpha =0.3,\;\varphi_{0}=0.$$

**Figure 3:**
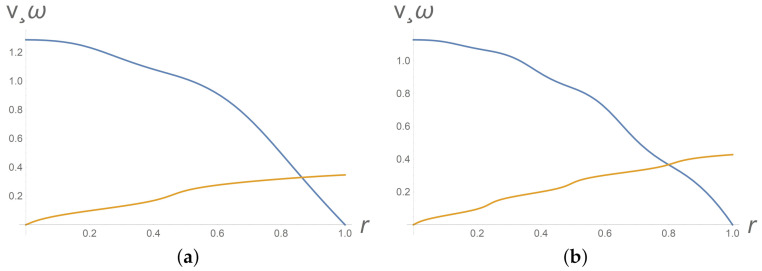
Gradient banding instability with respect to time. From top to bottom, dimensionless velocity v(y) and dimensionless micro-spin ω(y) profiles at Π=2.85 for different dimensionless times t=15 (**a**) and t=25 (**b**). Values of other parameters are as in the data list (29).

**Figure 4:**
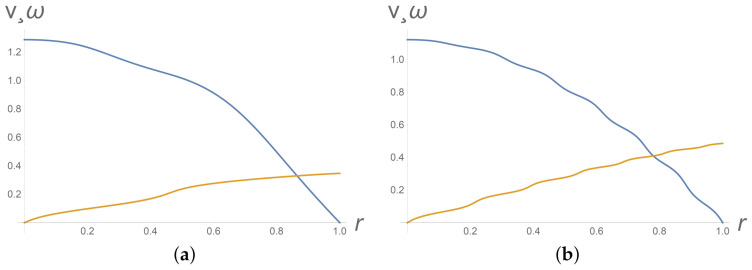
Gradient banding instability with respect to initial particles orientation. From top to bottom, profiles of dimensionless velocity v(y) and dimensionless micro-spin ω(y) at Π=2.85 and at t=15 for initial $\varphi_{0}\left(y\right)=0$ (**a**) and $\varphi_{0}\left(y\right)=4y+9y^{2}$ (**b**). Values of other parameters are as in the data list (29).

Intervals where φ(y)= const or ω(y)= const correspond to the nematic phase. The profiles of the intrinsic angular velocity ω at Figure 2b and Figure 3 imply appearance and instability of the nematic phase. Figure 4b depicts the phase transition from the isotropic phase to the nematic phase.

Figure 3 shows gradient banding instability with respect to time. A treatment of time dependent phenomena for worm-like micelles can be found in [5].

It turns out that the gradient banding is also unstable with respect to initial particles orientation. When passing from spatially homogeneous initial orientation of particles $\varphi_{0}\left(y\right)=0$ to a spatially heterogeneous orientation (like $\varphi_{0}\left(y\right)=$ 4y+ $9y^{2}$), the gradient banding effect becomes more pronounced, see Figure 4.

**Figure 5:**
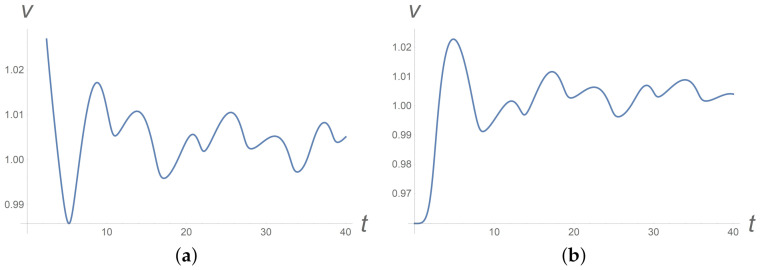
Time variation of the velocity in the middle of the channel at a constant pressure gradient in dimensionless variables (**a**) for homogeneous transversal initial particles orientation and (**b**) for non-homogeneous initial particles orientation along the channel.

Many shear banding systems display oscillations or irregular fluctuations. Example systems include worm-like micelles [37]. Within the developed anisotropic model, one can observe a chaotic behaviour of the shear velocity even at a constant applied pressure gradient, see Figure 5. Basically, it is due to anisotropic viscosities in the rheological constitutive laws (13).

Next, we consider questions motivated by oil transportation through pipelines. To optimize pumping, additives are used that change the microstructure of oil. As a result, it is discovered that friction factor can depend not only on oil discharge, but on its prehistory as well [38]. It turns out that the smallest friction losses are achieved by decreasing rather than increasing the flow rate to a predetermined level [9]. Let us show that the developed mathematical model in Section 1 captures such an a effect.

**Figure 6:**
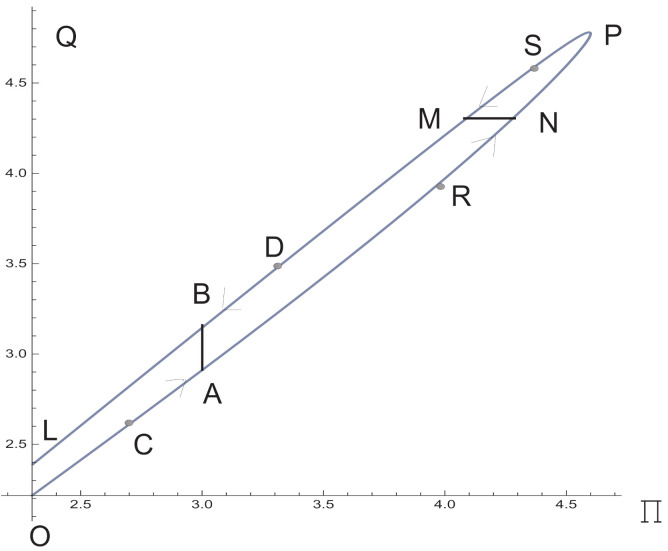
On the (Π,Q)-plane, the hysteresis loop corresponding to process (30) for rather small $\varepsilon_{20},\varepsilon_{30}$, with $\Pi_{0}=2.3$.

First, we establish that the system (26)–(28) does not provide one-to-one correspondence between the pressure gradient Π and the total fluid flux $Q=2\int_{0}^{1}v\;dy$. Given a time-dependent pressure gradient Π(t), one can calculate the corresponding total flux Q(t). Let us consider the parametric line

(30) $$\Pi =\Pi_{0}\left(1+\sin\pi t\right),\;Q=Q\left(t\right),\;0<t<1,$$

which corresponds to the curve $\hat{O,P,L}$ on the (Π,Q)-plane, Figure 6. The lower part $\hat{O,A,P}$ of this curve corresponds to the time interval 0<t<1/2. Along this part, both *Q* and Π grow, $\Pi_{0}<\Pi <2\Pi_{0}$. The top part $\hat{P,B,L}$ of the curve corresponds to the time interval 1/2<t<1. Along this part, both *Q* and Π decrease.

For typical viscous fluids like a power law fluid, there is a one-to-one correspondence between Π and *Q*; as a consequence, the lines $\hat{O,P,L}$ and $\hat{P,B,L}$ coincide. It is not the case for the anisotropic fluid considered here. Given $\Pi_{*}$ satisfying the inequalities $\Pi_{0}<\Pi_{*}<2\Pi_{0}$, how can one determine a corresponding flux *Q*? It follows from Figure 6 that there are two values $Q_{A}$ and $Q_{B}$ corresponding to $\Pi_{*}$. Indeed, let us consider the intersection of the vertical line $\Pi =\Pi_{*}$ with the curve $\hat{O,P,L}$. On this way we arrive at the points *A* and *B*:

$$A=\left(\Pi_{*},Q_{A}\right),\;B=\left(\Pi_{*},Q_{B}\right).$$

Clearly, there are $t_{A}$ and $t_{B}$ such that

$$0<t_{A}<1/2<t_{B}<1,\;\Pi \left(t_{A}\right)=\Pi \left(t_{B}\right)=\Pi_{*},\;Q_{A}<Q_{B},\;Q_{i}=Q\left(t_{i}\right),$$

with i=A,B.

Let us choose the points $C=(\Pi_{C},Q_{C})$ and $D=(\Pi_{D},Q_{D})$ in such a way that

$$\Pi_{C}<\Pi_{*}<\Pi_{D}.$$

When the value of Π goes from the low value $\Pi_{C}$ to $\Pi_{*}$, the value of *Q* changes from $Q_{C}$ to

$$Q_{A}=\lim_{\Pi ↗\Pi_{*}}Q\left(\Pi \right).$$

When the value of Π goes from the upper value $\Pi_{D}$ to $\Pi_{*}$, the value of *Q* changes from $Q_{D}$ to

$$Q_{B}=\lim_{\Pi ↘\Pi_{*}}Q\left(\Pi \right).$$

Thus, total flux depends not only on pressure gradient, but on the evolution history of pressure gradient as well.

Similarly, we consider determination of Π starting from values of *Q*. Again, one should know a prehistory of *Q*. Indeed, let us consider a total flux $Q_{*}$, which is between ${Q|}_{t=0}$ and ${Q|}_{t=1/2}$. Let us consider the intersection of the horizontal line $Q=Q_{*}$ with the hysteresis loop $\hat{O,P,L}$. In this way, we arrive at the points *N* and *M*:

$$N=\left(\Pi_{N},Q_{*}\right),\;M=\left(\Pi_{M},Q_{*}\right).$$

Clearly, there are $t_{N}$ and $t_{M}$ such that

$$0<t_{N}<1/2<t_{M}<1,\;Q\left(t_{N}\right)=Q\left(t_{M}\right)=Q_{*},\;\Pi_{M}<\Pi_{N},\;\Pi_{i}=\Pi \left(t_{i}\right),$$

with i=N,M. Let us choose points $R=(\Pi_{R},Q_{R})$ and $S=(\Pi_{S},Q_{S})$ in such a way that $Q_{R}<Q_{*}<Q_{S}.$

If *Q* goes from the lower value $Q_{R}$ to $Q_{*}$ then Π changes from $\Pi_{R}$ to

$$\Pi_{N}=\lim_{Q↗Q_{*}}\Pi \left(Q\right).$$

If *Q* goes from the upper value $Q_{S}$ to $Q_{*}$ then Π changes from $\Pi_{S}$ to

$$\Pi_{M}=\lim_{Q↘Q_{*}}\Pi \left(Q\right).$$

Thus, pressure gradient depends not only on total flux, but on the prehistory evolution of total flux as well.

As far as the oil pipelines are concerned, the designed oil flux can be obtained in two ways: by switching from a fast or slow flux. By the developed anisotropic model, the pressure drop to ensure the designed oil flux is less in the first case.

Now, we consider friction losses which play an important role in oil pumping through pipelines. Returning to dimension variables, we remind that the mean velocity *U* and the friction factor λ are defined as follows:

$$U=\frac{1}{2H}\int_{-H}^{H}v\left(y\right)\;dy,\;\left|p_{x}\right|=\frac{\lambda }{2H}\frac{\rho U^{2}}{2}.$$

In dimensionless variables, we have

$$U^{\prime }=\int_{0}^{1}v^{\prime }\left(y\right)dy=\frac{Q}{2},\;\Lambda \equiv \frac{Re\cdot \lambda }{8}=\frac{\Pi }{U^{\prime }{}^{2}},$$

where Λ is the reduced friction factor.

**Figure 7:**
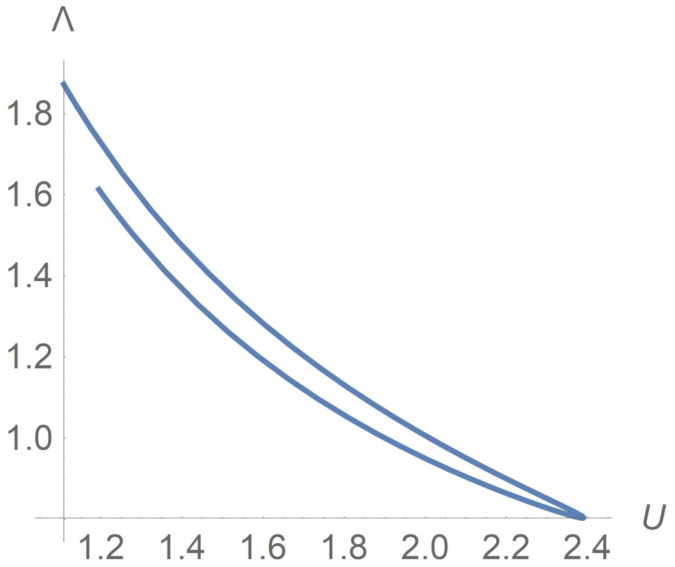
Hysteresis loop on the (U,Λ) plane, where *U* is the mean velocity and Λ is the friction factor. The data are the same as in Figure 2.

To analyse flows on the plane $\left(U^{\prime },\Lambda \right)$, we omit the prime indexes. Calculations reveal that, starting from the pressure gradient law

$$\Pi \left(t\right)=\Pi_{0}\left(1+\sin\pi t\right),$$

the curve

$$U=U\left(t\right),\;\Lambda \left(t\right)=\frac{\Pi (t)}{U^{2}\left(t\right)},\;0<t<1,$$

becomes as is shown in Figure 7. The top part of this curve corresponds to the time interval 0<t<1/2. Along this part, both *U* and Π grow, $\Pi_{0}<\Pi <2\Pi_{0}$, whereas Λ decreases. The lower part of the curve corresponds to the time interval 1/2<t<1. Along this part, both *U* and Π decrease, whereas Λ grows. How can one calculate the friction factor Λ corresponding to a designed mean velocity $U^{*}$? The answer depends on the history; one can attain $U_{*}$ by increasing *U* or by decreasing *U*. Given $U_{*}$ lying between $U_{min}{=U|}_{t=0}$ and $U_{max}{=U|}_{t=1/2}$, we choose $t_{1}$ and $t_{2}$ in such a away that

$$0<t_{1}<1/2<t_{2}<1,\;U\left(t_{1}\right)=U\left(t_{2}\right)=U_{*}.$$

With $\Lambda_{i}$ standing for $\Lambda (t_{i})$, one can conclude from Figure 7 that $\Lambda_{1}>\Lambda_{2}$ despite the fact that both $\Lambda_{1}$ and $\Lambda_{2}$ correspond to the same $U_{*}$. Thus,

$$\Lambda_{1}=\lim_{U↗U_{*}}\Lambda \left(U\right)=\Lambda {{|}_{U_{*}-}>\Lambda |}_{U_{*}+}=\lim_{U↘U_{*}}\Lambda \left(U\right)=\Lambda_{2}.$$

Returning to the issue of oil transportation, one can attain the productive regime in two ways by switching from a faster or from a slower flux. After switching to a productive regime, the developed friction loss is less in the first case. Such a conclusion agrees with known in situ data [9].

Consider the stress response to a change in velocity gradient. It follows from the dimensional steady-state Equation (22) that the shear stress $S_{12}$ is given by the formula

$$S_{12}=p_{x}y,\;\tilde{\tau }\equiv -S_{12}{|}_{y=H}=-p_{x}H,$$

where $\tilde{\tau }$ is the stress at the upper plane y=H. Let us calculate the curve $\tilde{\tau }=\tilde{\tau }\left({\dot{\gamma }}_{1}\right)$ where ${\dot{\gamma }}_{1}=-v_{y}{|}_{y=H}$. Observe that ${\dot{\gamma }}_{1}$ does not stay for the the shear rate in the micropolar fluid theory. We pass to the dimensionless variables

$$\tau =\frac{\tilde{\tau }}{2\mu_{s}\Omega }=\Pi ,\;\dot{\gamma }=-v_{y^{\prime }}^{\prime }{|}_{y^{\prime }=1},\;{\dot{\gamma }}_{1}=\Omega \dot{\gamma }.$$

**Figure 8:**
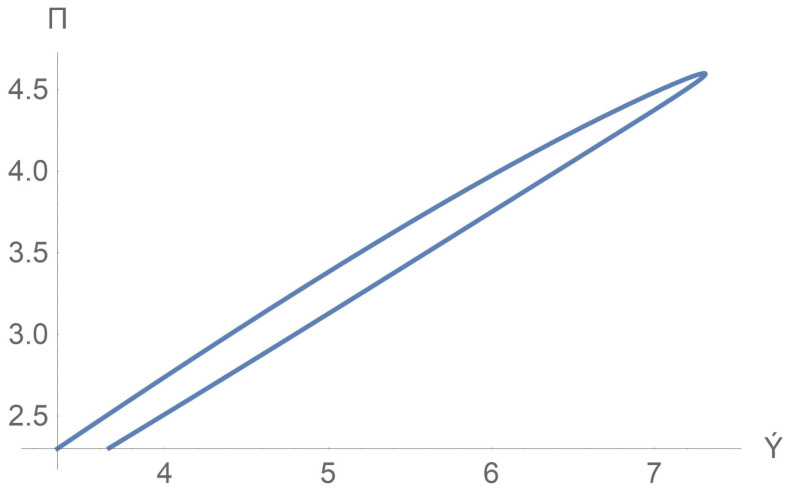
Hysteresis of the rheological stress–strain curve $\Pi =\Pi (\dot{\gamma })$.

Performing calculation of the parametric curve

(31) $$\Pi =\Pi_{0}\left(1+\sin\pi t\right),\;\dot{\gamma }=\dot{\gamma }\left(t\right),\;0<t<1,$$

we arrive at the hysteresis loop $\tau =\tau (\dot{\gamma })$, which is shown in Figure 8. Thus, there is no one-to-one correspondence between velocity gradient and shear stress in shear flows. Such an effect has been seen in worm-like micelle solutions [39].

**Figure 9:**
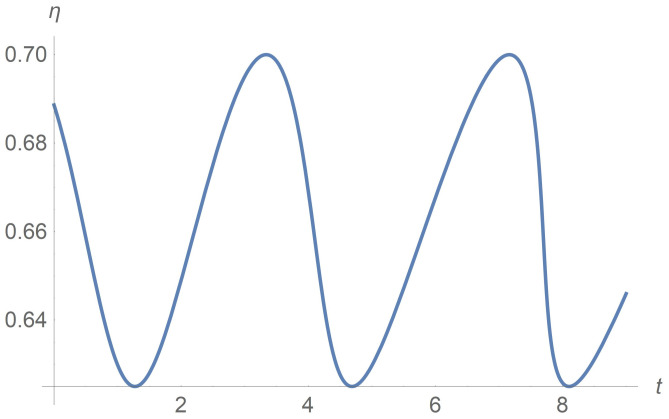
Apparent viscosity versus time. The case $\Pi =\Pi_{0}=\mathrm{const}$.

Let us introduce the apparent viscosity $\eta_{a}=\tilde{\tau }/{\dot{\gamma }}_{1}$. Figure 9 depicts how its dimensionless replica $\eta =\tau /\dot{\gamma }$ varies with time for the case Π=const. Sustained temporal oscillations of macroscopic viscosity are observed in [40] for the rod-like suspension.

**Figure 10:**
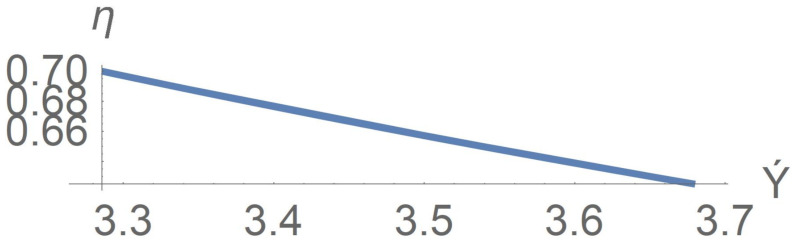
Apparent viscosity η versus velocity gradient $\dot{\gamma }$ for the case $\Pi =\Pi_{0}=\mathrm{const}$.

As far as the function $\eta =\eta (\dot{\gamma })$ is concerned, the shear thinning nature of suspensions of rod-like particles is clearly depicted on Figure 10 in agreement with observations in [1].

## 5. Discussion

We address rheology of suspensions of rodlike particles. To take into account both particle–fluid and particle–particle interactions, we treat the suspension as a Cosserat continuum and apply the micropolar fluid theory approach. Assuming that local stress depends on the rods directions, we include the micro-inertia tensor into constitutive laws as an independent variable jointly with the rate of strain tensors. The micropolar fluid theory allows for particle’s rotation obeying the angular momentum conservation law. The Cauchy stress tensor loses symmetry and the couple stress tensor is of importance. This is why one should formulate two stress-rate of strain rheological equations for the stress tensor and the couple stress tensor. Unlike a Newtonian fluid, a micropolar fluid is characterized by two rates of strain tensors, through which the linear velocity gradient and the angular velocity gradient are expressed. The impact of variation of rate of strains and the micro-inertia onto the local stress state in the rheological equations is manifested through the viscosities. This is why, in addition to the usual shear viscosity, we also introduce skew-symmetric and anisotropic viscosities. The derived governing equations are proved to be consistent with basic principles of thermodynamics. By performing calculations of simple one-dimensional pressure driven flows between two parallel planes, we establish that the skew-symmetric and the anisotropic viscosities underlie a number of important properties, which include gradient banding instability, coexistence of isotropic and nematic phases, sustained temporal oscillations of macroscopic viscosity, shear thinning and hysteresis. Keeping in mind data for oil transport in pipelines, we also establish that the total flow rate depends not only on the pressure gradient, but on the history of its variation as well.

## Data Availability

Not applicable.
